# Supplementary material for: Antibiotic Resistance of Staphylococcus aureus Strains—Searching for New Antimicrobial Agents—Review
Source: Pharmaceuticals (Basel). 2025 Jan 11;18(1):81. doi: 10.3390/ph18010081 (PMC11768290; doi:10.3390/ph18010081)
Supplement: Supplementary file 1 [file pharmaceuticals-18-00081-s001.zip › pharmaceuticals-3335857-supplementary.pdf]

pharmaceuticals

MDPI

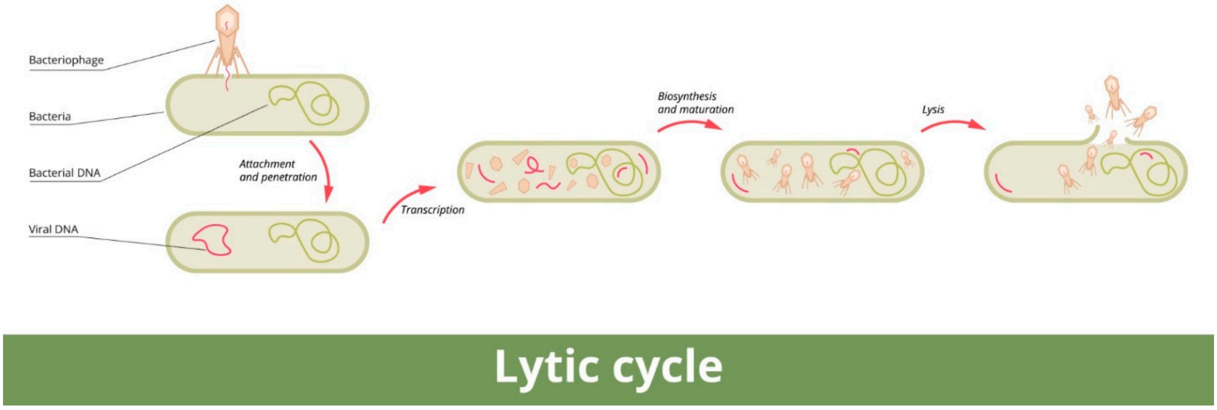

Figure S1. Phage lytic cycle. (https://as2.ftcdn.net/v2/jpg/05/17/17/89/1000_F_517178920_xvAcJJmMWkuDRmOI6secnihiLybQvHyf.jpg).

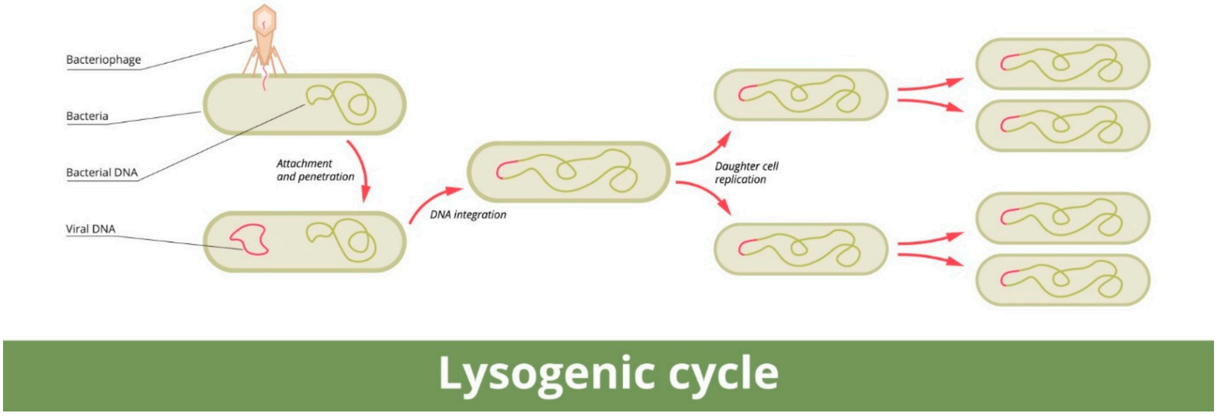

Figure S2. Phage lysogenic cycle. (https://stock.adobe.com/pl/images/lysogenic-cycle-the-viral-reproduction-cycle-is-characterized-by-integrating-the-bacteriophage-nucleic-acid-into-the-host-bacterium-s-genome-or-forming-a-circular-replicon-in-the-bacterial-cytoplasm/517178917).
